# Enhancement of Superconductivity by Amorphizing Molybdenum Silicide Films Using a Focused Ion Beam

**DOI:** 10.3390/nano10050950

**Published:** 2020-05-16

**Authors:** Emma Mykkänen, Arijit Bera, Janne S. Lehtinen, Alberto Ronzani, Katja Kohopää, Teresa Hönigl-Decrinis, Rais Shaikhaidarov, Sebastian E. de Graaf, Joonas Govenius, Mika Prunnila

**Affiliations:** 1 VTT Technical Research Centre of Finland Ltd, P.O. Box 1000, FI-02044 VTT Espoo, Finland; arijit.bera@vtt.fi (A.B.); janne.lehtinen@vtt.fi (J.S.L.); alberto.ronzani@vtt.fi (A.R.); katja.kohopaa@vtt.fi (K.K.); joonas.govenius@vtt.fi (J.G.); mika.prunnila@vtt.fi (M.P.); 2 National Physical Laboratory, Hampton Road, Teddington TW11 0LW, UK; teresa.hoenigl-decrinis@npl.co.uk (T.H.-D.); sebastian.de.graaf@npl.co.uk (S.E.d.G.); 3 Royal Holloway, University of London, Egham TW20 0EX, UK; R.Shaikhaidarov@rhul.ac.uk; 4 Moscow Institute of Physics and Technology, 141700 Dolgoprudny, Russia

**Keywords:** superconductivity, molybdenum silicide, focused ion beam

## Abstract

We have used focused ion beam irradiation to progressively cause defects in annealed molybdenum silicide thin films. Without the treatment, the films are superconducting with critical temperature of about 1 K. We observe that both resistivity and critical temperature increase as the ion dose is increased. For resistivity, the increase is almost linear, whereas critical temperature changes abruptly at the smallest doses and then remains almost constant at 4 K. We believe that our results originate from amorphization of the polycrystalline molybdenum silicide films.

## 1. Introduction

Amorphous atomic structure for conductive thin films is desirable when the spatial homogeneity down to the nanoscale is of the essence for device functionality. This is the case, for example, for superconducting nanowire single photon detectors (SNSPD) [1,2,3] where inhomogeneities create local weak spots, leaving the rest of the nanowire under-biased. Homogeneity is also essential for quantum phase slip (QPS) components [4] where the phase tunneling rate depends exponentially on the geometric uniformity of the nanowire.

A typical way to make amorphous thin films is by sputtering on cooled surfaces [5]. Amorphous films have also been made with other techniques including e.g., liquid quenching [6], neutron bombardment [7] and ion-mixing [8]. Here, we utilize a simple post-processing method: bombarding the sample with ions using a focused ion beam (FIB). This technique can be applied locally with nanometer scale spatial resolution to tune normal state resistivity, superconducting transition temperature, and critical current. In the large dose limit, local weak spots can be created, which was also recently demonstrated with cuprates [9] and niobium nitride [10]. Remarkably, we also observe the opposite at lower doses, i.e., enhancement of superconductivity under moderate exposure to a FIB.

In this article, we use a gallium FIB to amorphize thermally formed molybdenum silicide (MoSi). The superconducting properties of this material are strongly dependent on its fabrication process [11,12]. Its applications range from CMOS processes for quantum circuits [13] to SNSPD and QPS operation [5,14,15]. In addition, silicides have been shown to exhibit excellent microwave performance when fabricated with diffusion barriers [16,17].

In this paper, we demonstrate that the resistivity of MoSi depends almost linearly on the FIB dose and that the critical temperature increases from about 1 K to 4 K when the samples are irradiated with ions. We believe that our results originate from the amorphization of MoSi instead of doping the nanowires with gallium. This is supported by independent tests with a helium FIB, since an inert gas like helium should not introduce doping. However, the gallium FIB is our main workhorse due to its higher mass that reduces the patterning time of amorphous nanowires.

## 2. Materials and Methods

**Figure 1:**
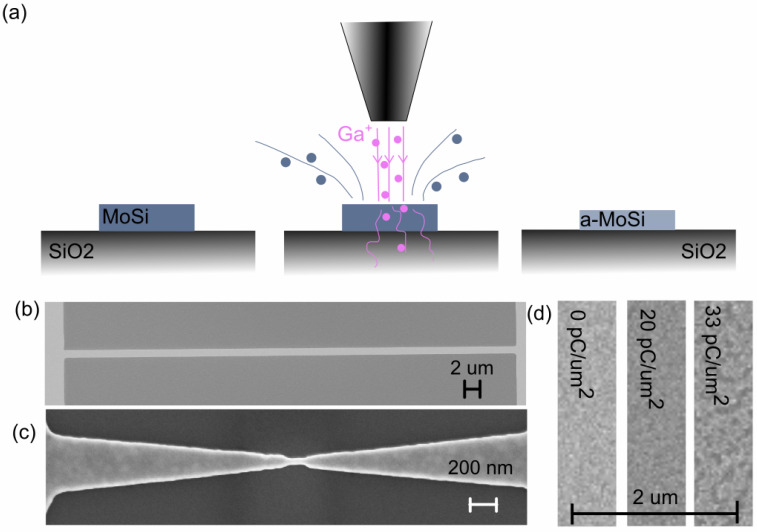
(**a**) Conceptual image of the irradiation of MoSi using focused ion beam. (**b**) Scanning electron micrograph showing the geometry for micrometer scale wires. (**c**) Scanning electron micrograph showing the geometry for nanometer scale samples. (**d**) Scanning electron micrographs with different FIB doses showing that surface roughness increases at doses larger than approximately 20 pC/μm${}^{2}$, but no visible effect is seen below that. We identify this dose as a threshold for significant film ablation.

We fabricate the samples on top of silicon oxide by sequentially sputtering molybdenum and silicon and then annealing at 600 °C. After patterning, the MoSi is irradiated with a gallium ion beam of the FEI Helios Nanolab 600 dual beam system (Figure 1a). The ion beam acceleration voltage and current are 30 kV and either 2.8 nA or 21 nA. More details on sample fabrication can be found in Appendix A.

We utilize two types of samples as shown in Figure 1b,c. The micrometer scale wires (Figure 1b) are used to study the effect of FIB in a wide parameter range. Since earlier results [15] show that the superconducting behavior may change depending on sample size, we also investigate nanometer scale structures (Figure 1c).

In most of the samples, the FIB is directly allowed to mill the MoSi, which results in slight thinning of the structures. The milling is uniform and we observe no surface damage up to doses of about 20 pC/μm${}^{2}$ which is already several times higher than the typical dose used for the amorphisation (see Figure 1d). Only at doses above 30 pC/μm${}^{2}$ degradation begins, likely due to uneven sputter etching of the film surface (see Figure 1d). The uniform milling is taken into account in the analysis (see Appendix B). However, in general, it would be desirable to control the milling and amorphization effects separately. To study this, we fabricated samples that have a layer of oxide protecting the MoSi from the FIB. These samples have a geometry similar to the uncoated micrometer scale wires (see Figure 1b).

The proportion of molybdenum to silicon affects the critical temperature of the compound [5]. The lower the silicon content, the higher the critical temperature [5]. On the other hand, if the silicon content is below approximately 25% there is possibility of spontaneous amorphous to crystalline transition due to build up of tensile stress [12]. Critical temperatures as high as 7.5 K have been obtained for amorphous MoSi with stoichiometry of 3 Mo per 1 Si [14] and we choose this as our starting point. The final thickness values of our films are 10 nm of molybdenum and 6 nm of silicon for the uncoated wires (both micrometer and nanometer scale) and 10 nm of molybdenum to 5 nm of silicon for the coated wires. These correspond to a stoichiometry of Mo${}_{69}$Si${}_{31}$ and Mo${}_{72}$Si${}_{28}$, respectively (see Appendix A). The thickness estimates are obtained from calibrated sputtering rates.

## 3. Results and Discussion

**Figure 2:**
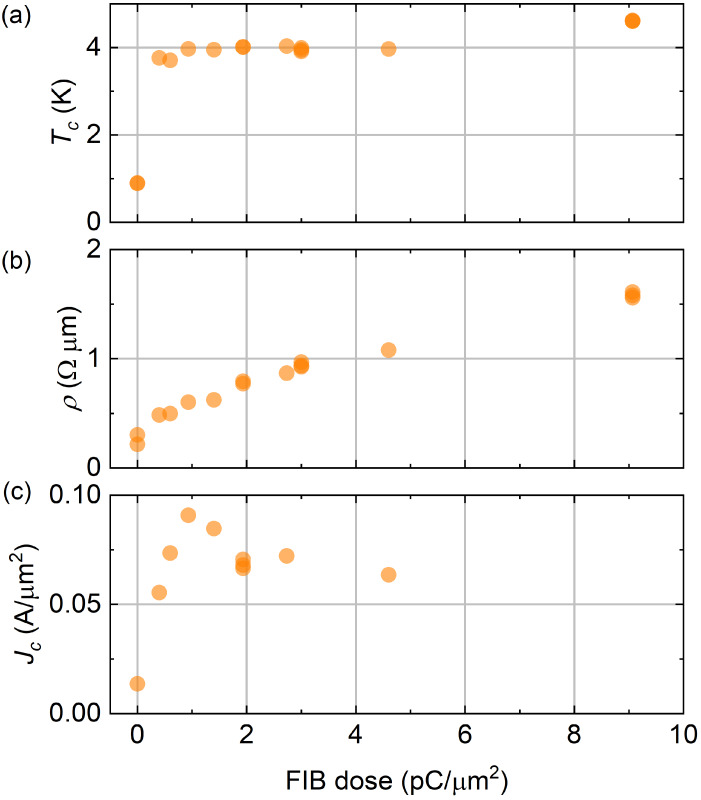
The effect of FIB dose on (**a**) critical temperature, (**b**) normal state resistivity, and (**c**) critical current density of the uncoated micrometer scale samples (Figure 1b). The milling caused by the FIB is taken into account in the analysis.

Figure 2 shows the critical temperature, $T_{c}$, low temperature normal state resistivity, ρ, and critical current density, $J_{c}$, as functions of FIB dose for uncoated micrometer scale samples (for more details on measurements see Appendix B). We observe that the critical temperature undergoes an abrupt transition from 0.9 K to 3.8 K with the smallest FIB dose (around 0.33 pC/μm${}^{2}$), after which it stays approximatively constant. On the other hand, the resistivity scales almost linearly as a function of the dose. These two features enable tunability of the resistivity (and thus kinetic inductance) independent of $T_{c}$. In applications at intermediate temperatures (between 1 K and 3 K), the sharp transition in critical temperature between untreated and treated wires also allows convenient spatially selective formation of resistors without additional processing steps. The critical current density of our samples varies between 10 and 100 mA/μm${}^{2}$ and peaks at a dose of about 1 pC/μm${}^{2}$.

The results of Figure 2 suggest that MoSi undergoes a transition to a more amorphous phase when it is bombarded by gallium ions. In previous studies, the superconductivity of MoSi has been observed to be nontrivially dependent of the multi or single layer composition of MoSi as well as its crystalline or amorphic properties [11]. However, the simultaneous increase of resistivity and enhancement of superconductivity is a clear indication of amorphization. In fact, amorphization increases static disorder which increases resistance and electron-phonon coupling which enhances critical temperature in BCS superconductors [18]. The enhancement of superconductivity also explains the initial increase of critical current density. Its subsequent decrease at higher doses may be attributed to excess defect formation and eventual film ablation.

**Figure 3:**
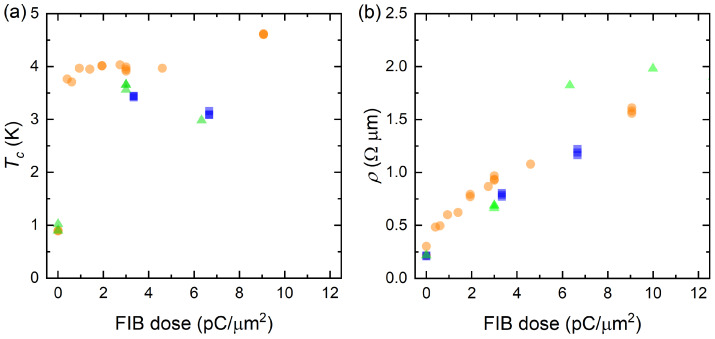
Critical temperature (**a**) and normal state resistivity (**b**) as a function of FIB dose for the uncoated micrometer scale wires (orange circles, see Figure 1b), coated micrometer scale wires (blue squares, see Figure 1b) and nanometer scale samples (green diamonds, see Figure 1c). The milling caused by the FIB is taken into account in the analysis.

Amorphization is beneficial for example in devices where spatial homogeneity down to nanoscale is of essence. This is the case for example with SNSPD and QPS components. Based only on the data in Figure 2, one may question whether the superconductivity is enhanced homogeneously enough to also apply to narrow nanowires, which are of significant practical interest for these components. To this end, Figure 3 compares the micrometer scale samples to much smaller wires, demonstrating comparable results (for more info on measurements see Appendix B). However, to form definite conclusions, further investigation of atomic scale structure of our samples would be needed for example with transmission electron micrography or diffraction peak spectroscopy.

Directly irradiating our samples with FIB causes no more than 10% reduction in film thickness (which is taken into account in the analysis, see Appendix B), but on the other hand, adding a protective oxide may change the penetration profile of gallium ions in the MoSi layer. Figure 3 shows that adding the oxide on top does not change drastically the overall behaviour of critical temperature or normal-state resistivity, making it a valid option for protecting the film. The deviations of critical temperature may be attributed to different density profile of defects introduced in the MoSi layer, and possibly to variations in its stoichiometry due to silicon atoms displaced from the coating.

**Figure A1:**
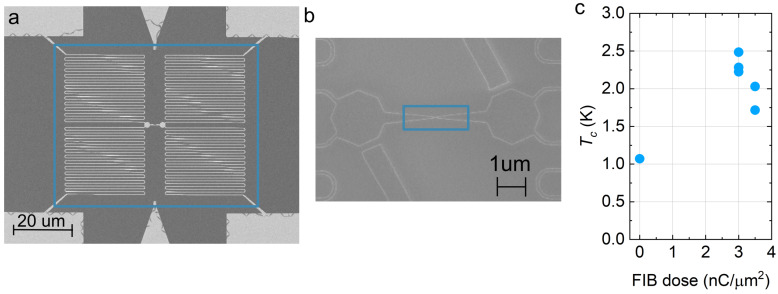
(**a**) Large scale image of a sample. The light blue square indicates the area exposed by gallium FIB. (**b**) Closeup on the sample showing also the area exposed when studying effect of helium FIB (light blue square). (**c**) Critical temperature as a function of helium FIB dose.

In experiments where gallium beam has been used to etch structures, gallium poisoning has also been an issue [19]. In order to rule out the possibility of doping being the dominant mechanism explaining our results, we also investigated how replacing gallium by helium affects the results (see Appendix C). These tests yield an enhancement in critical temperature from the initial value of 1 K of untreated wires to around 1.5–2.5 K when the helium FIB is applied (see Figure A1c). This behavior is analogous to gallium, although the obtained critical temperatures are smaller. The differences may be attributed to the different interaction profiles of helium and gallium, as well as the small size of the area treated by the helium FIB and the proximity effect caused by weakly conducting surroundings (see Appendix C for details). The proximity effect is present also in samples irradiated by gallium, but due to the heavier mass of gallium atoms and subsequently faster writing times, we could expose areas much larger than the lateral length scale of the proximity effect, making its contribution negligible.

## 4. Conclusions

In conclusion, we have used a gallium-based focused ion beam to tune the superconducting parameters of MoSi. Irradiating MoSi with gallium causes its critical temperature to rise from an initial value of 1 K to about 4 K after bombardment. This change is abrupt occurring even at a dose as low as 0.5 pC/μm${}^{2}$. In addition, the resistivity shows a gradual monotonic increase as a function of dose, while the critical current density of our samples varies nonmonotonically between 10 and 100 mA/μm${}^{2}$. These results allow tunability of resistivity (and thus kinetic inductance), as well as operation at temperatures suitable for simple pulse tube cryocoolers. We believe that our results originate from the amorphization of MoSi. This is supported by independent measurement using samples treated with helium FIB.

## Abbreviations

The following abbreviations are used in this manuscript:

FIB	focused ion beam
MoSi	molybdenum silicide
QPS	quantum phase slip
SNSPD	superconducting nanowire single photon detector
CMOS	complementary metal-oxide-semiconductor
HSQ	hydrogen silsesquioxane

## Appendix A. Sample Fabrication

As a substrates we use 6 inch <100>-type silicon wafers with p-type phosphorus doping and resistivities of about 1–50 Ωcm. The substrate is isolated from the molybdenium silicide by thermal silicon oxide, which has a thickness of either 1000 nm (capped micrometer scale samples) or 10 nm (uncapped micrometer scale samples). Two different wafers are used for the nanometer scale samples and these have oxide thickness values of 10 nm and 1000 nm, respectively.

The oxide growth is followed by sputter deposition of 10 nm of molybdenum and 3 to 8 nm of silicon. To pattern our structures, the wafer is coated with a negative photoresist HSQ, baked at 150 °C, and subsequently patterned by electron beam lithography and an optical stepper. The unexposed areas of Mo and Si are removed by etching. Depending on the sample, the HSQ is either removed by an HF dip or left in place to form a protective oxide coating. The HF dip also removes some amount of silicon. The final thickness values are presented in Table A1. To form the molybdenum silicide the samples are annealed at 600 °C for 10 min.

**Table A1:** Amount of molybdenium and silicon before annealing, average number of molybdenium atoms per silicon atoms in the compound, and presence or absence of protective oxide above MoSi when exposed to FIB. The proportion of molybdenum atoms to silicon atoms is calculated from the material thickness values using textbook values for density and molar mass. The thickness values are obtained from calibrated sputtering rates.

Samples	Mo (nm)	Si (nm)	Stoichiometry	Oxide on Top
uncoated micrometer scale wires	10	6	69 Mo per 31 Si	no
nanometer scale samples	10	6	69 Mo per 31 Si	no
coated micrometer scale wires	10	5	72 Mo per 28 Si	HSQ 20 nm

Finally, the samples are irradiated with the focused ion beam of FEI Helios Nanolab 600 dual beam system. The ion beam has acceleration voltage of 30 kV and we use either 2.8 nA or 21 nA current. For uncoated samples we add 1 μm blur for the focused ion beam and use a pitch of 125 μm. The blur is not utilized when patterning samples with the protective HSQ layer.

## Appendix B. Measurement Setup

We utilize either a lock-in technique with small excitation frequency (between 1 Hz and 30 Hz), or fast IV sweeps to measure the temperature dependence of resistivity. This data is used to obtain the normal state resistivity and critical temperature. The normal state resistivity is recorded at the temperature just before the transition starts and we define the critical temperature as the temperature, where resistivity falls to half of its normal state value. The critical current densities are measured at the base temperature of a Bluefors cryostat, which was around 10 mK. Due to the large size of the wires, the critical currents are relatively large, around 1 mA, which causes peak heating of the order of milliwats after switching to resistive state. However, we see no evidence that such heating would have changed the properties of the nanowires. For example, switching to normal state did not introduce smearing of the IV curves or residual voltage in the superconducting state.

The etching caused by the FIB can decrease both the thickness and width of the samples, and this is taken into account in the analysis. The widths of the samples are obtained from SEM images. We use the value 0.15 μm${}^{3}$/nC for the vertical etching rate. This value is obtained from the manual of FEI Helios Nanolab 600 for etching silicon, which we believe is close enough to give us a rough estimate of the etching rate.

## Appendix C. Effect of Helium FIB

To rule out effects related to gallium poisoning, we irradiate samples using a helium FIB. Otherwise the fabrication follows the same procedure as with the gallium FIB (see Appendix A), except that the samples have initial silicon thickness of 3 nm and a stoichiometry of 81 Mo per 19 Si. In addition, we add a protective PECVD layer to the wafer after annealing, but before irradiation with helium. To irradiate the samples, we utilize the FIB in a Zeiss Orion NanoFab Helium ion microscope and use an acceleration voltage of 12.9 kV and current of 60 pA.

Since helium is an inert gas with a light mass, larger doses are needed than with gallium, and thus the exposure times increase. Therefore, we expose substantially smaller areas of around 1 μm${}^{2}$ (see Figure A1a,b). Figure A1c shows the results of the helium FIB. We see an abrupt enhancement of critical temperature similar to gallium, but the obtained values are lower than their gallium counterparts. The most likely explanations are the different interaction profile of the two ions and the proximity effect induced by the untreated surroundings of the small area treated with helium. We would also like to note that the stoichiometry of the samples treated with the helium FIB is different from that of the samples treated with gallium. Stoichiometry has been shown to affect the critical temperature of MoSi, although decreasing silicon content should increase the critical temperature, not decrease it as is in our case [5].
